# Heterochromatin is a quantitative trait associated with spontaneous epiallele formation

**DOI:** 10.1038/s41467-021-27320-6

**Published:** 2021-11-29

**Authors:** Yinwen Zhang, Hosung Jang, Rui Xiao, Ioanna Kakoulidou, Robert S. Piecyk, Frank Johannes, Robert J. Schmitz

**Affiliations:** 1 Institute of Bioinformatics, University of Georgia, Athens, GA USA; 2 Department of Genetics, University of Georgia, Athens, GA USA; 3 Department of Plant Sciences, Technical University of Munich, Freising, Germany; 4 Institute for Advanced Study (IAS), Technical University of Munich, Garching, Germany

**Keywords:** DNA methylation, Chromatin structure, Plant molecular biology

## Abstract

Epialleles are meiotically heritable variations in expression states that are independent from changes in DNA sequence. Although they are common in plant genomes, their molecular origins are unknown. Here we show, using mutant and experimental populations, that epialleles in *Arabidopsis thaliana* that result from ectopic hypermethylation are due to feedback regulation of pathways that primarily function to maintain DNA methylation at heterochromatin. Perturbations to maintenance of heterochromatin methylation leads to feedback regulation of DNA methylation in genes. Using single base resolution methylomes from epigenetic recombinant inbred lines (epiRIL), we show that epiallelic variation is abundant in euchromatin, yet, associates with QTL primarily in heterochromatin regions. Mapping three-dimensional chromatin contacts shows that genes that are hotspots for ectopic hypermethylation have increases in contact frequencies with regions possessing H3K9me2. Altogether, these data show that feedback regulation of pathways that have evolved to maintain heterochromatin silencing leads to the origins of spontaneous hypermethylated epialleles.

## Abstract (web summary)

The molecular origins of epialleles remain unknown. Here, the authors show that a positive feedback loop between H3K9me2 and CMT2/3 is a major contributing factor to the origins of spontaneous epialleles and that heterochromatin is a quantitative trait associated with spontaneous epiallele formation.

## Introduction

Genetic variation is the primary driver of phenotypic variation, yet, there are well-documented examples of phenotypes arising independent of changes to the DNA sequence. These variants are referred to as epigenetic alleles (epialleles) and they can be transmitted across generations, especially in plants^1,2^. However, the extent and the contribution of natural epialleles underlying certain traits is less clear^3^. This lack of knowledge is mostly a consequence of the challenges to studying epialleles, both in their identification and in demonstrating causality.

The best characterized epialleles causally linked to phenotypes were discovered in plants and most were associated with changes in DNA methylation^4–9^. For example, *colorless non-ripening* (*cnr*) epiallele results from spontaneous formation of DNA methylation in its upstream regulatory region and a decrease in gene expression, resulting in tomato fruits with a mealy discolored skin compared to wild type^5^. Once formed, these particular epialleles are stably inherited in subsequent generations, with rare reversion events observed along the way. The use of whole genome bisulfite sequencing (WGBS)^10,11^ to identify differentially methylated regions has rapidly advanced our understanding of epiallele frequency, stability and molecular nature. For example, WGBS of a population of mutation accumulation lines of *Arabidopsis thaliana* shows that epialleles are rare, enriched in transcribed regions (genes, repeats and transposons) and are relatively stable once formed^12–14^. Importantly, the single base resolution nature of WGBS also reveals which DNA methylation pathways are most often associated with epiallele formation^10,11^.

In plants, cytosine DNA methylation is present at CG, CHG (H = A, C or T) and CHH sites^15^. Multiple independent pathways/enzymes coordinately reinforce DNA methylation, which ensures its stability through mitotic and meiotic cell divisions. For example, METHYLTRANSFERASE 1 (MET1) is recruited to hemimethylated CGs during S-phase of DNA replication to maintain their methylation^16,17^. Upon exiting S-phase, CHROMOMETHYLASE2 and 3 (CMT2/3) are recruited to DNA associated with histone 3 lysine 9 dimethylation (H3K9me2) to methylate CWA (W = A or T) and CHG sites, respectively^18–21^. After the cell exits the cell cycle, the RNA-directed DNA methylation (RdDM) pathway, which is guided by small RNAs, directs DOMAINS REARRANGED METHYLTRANSFERASE 2 (DRM2) to methylate cytosines in any sequence context^22^. Often, these pathways are found acting upon the same sequences and their coordinated efforts result in the relatively stable DNA methylation patterns that are observed between distinct cell types and over generational timescales in plants^23^.

Although epialleles are readily identified and characterized using WGBS to detect losses and gains of DNA methylation, their spontaneous origins are unknown. This has led to use experimental approaches to follow that fate of epialleles and to increase the frequency of epiallele formation to study their molecular origins and their association with traits. For example, silenced epialleles can be induced at a family of *MuDR* transposons in maize by exposing them to a silencing trigger *Muk*^24^. Although *MuDR* elements are silenced after exposure to a silencing trigger, one specific *MuDR* element reverts over time in the absence of the trigger. Other examples of experimental induced epialleles includes the creation of epigenetic recombinant inbred lines (epiRILs) in *Arabidopsis thaliana*^25,26^, whereby RILs are created between wild type and a mutant defective in maintenance of DNA methylation (*met1* or *DECREASE IN DNA METHYLATION 1—ddm1*)^27,28^. The epialleles in the epiRIL populations are mostly due to losses or gains of parental methylation states, however, epialleles also form at novel regions that were not present in either parent. The molecular basis for the origins of epialleles is an active area of investigation and there are some clues from previous published studies. For example, the creation of hybrids between *met1* and wild type reveals widespread redistribution of DNA methylation^29^. Wild-type chromosomes experienced hypomethylation, which was complemented by greater methylation on the *met1* chromosomes^29^. Moreover, a comparison of the DNA methylomes between 1st generation *ddm1* mutants and a 9th generation *ddm1* mutants shows increasing reductions of DNA methylation over generations^30^. Unexpectedly, novel epialleles, in the form of ectopic DNA methylation, are abundant in the 9th generation mutants even though these plants are essentially wild type in sequence^30^. These data point to a model whereby epigenomes are maintained by some unknown mechanisms that involves some level of feedback regulation based on the genomes ability to sense its overall epigenomic state.

There are multiple examples of feedback regulation of epigenomic states in plants. For example, expression of the DNA demethylase *REPRESSOR OF SILENCING 1* (*ROS1*) is sensitive to genome-wide levels of DNA methylation^31^. Similarly, the expression of the full-length transcript encoding an H3K9 demethylase, named *INCREASE IN BONSAI METHYLATION 1* (*IBM1*)^32^, is sensitive to H3K9me2/DNA methylation levels within an intronic repeat^33^. Additional clues of epigenomic feedback regulation are provided by increasing phenotypic variation with each additional generation of selfing in the *Arabidopsis thaliana* mutants *met1, ddm1* and *ibm1*^27,30,32,34^.

Evidence for DNA methylation feedback regulation is even observed in natural populations of *Arabidopsis thaliana* where a negative correlation was found between the abundance of CHG methylation, and the frequency of gene body DNA methylated (gbM) genes^35^. This leads to a model whereby the abundance of heterochromatin, demarcated by H3K9me2 and CHG methylation, is linked to the origins of epialleles that transition from unmethylated to gbM genes^35^. However, the natural variation in gbM is dependent on a functioning *CMT3* pathway, as without *CMT3*, gbM cannot be established and maintained^36–38^.

Although evidence for DNA methylation feedback regulation between chromosomes and its role in epiallele formation is mounting^39^, there is still a lack of mechanistic understanding of this process. How is it that unmethylated sequences become newly methylated spontaneously? What provides the trigger and why are some regions more susceptible for epiallele formation than others? In this study, we follow the fate of epialleles in genes that are either converted from unmethylated to methylated or from gbM to a transposon like methylation (teM) pattern in a variety of experimental mutant lines and populations. We show that loss of *ibm1* leads to ectopic CMT2 and 3 activity in the form of CWA and CHG methylation, respectively, and almost exclusively at gbM genes compared to unmethylated genes. This ectopic activity also occur, albeit at a lower frequency, when methylation of these gbM genes is erased using a *met1* epiRIL line. This shows the importance of the CMT3-H3K9me2 feedback loop in the establishment of epialleles. This is further evaluated using base resolution methylomes of 169 *ddm1*-derived epiRILs, where the abundance of heterochromatin is a variable trait in these lines. Using these data, we discover that variation in the genome-wide levels of CHG methylation, which serves as a proxy for heterochromatin, is negatively correlated with the abundance of epiallele formation, most of which is targeted to gbM genes. Methylation QTL analysis further confirm the importance of heterochromatin for epiallelic variation, as pericentromeres are hotspots for QTL. Collectively, this study demonstrates that a positive feedback loop between H3K9me2 and CMT2/3 is a major contributing factor to the origins of spontaneous epialleles and that heterochromatin is a quantitative trait that influences epiallele formation.

## Results

### Ectopic hypermethylation accumulates in gbM genes of methylation mutants

**Fig. 1:**
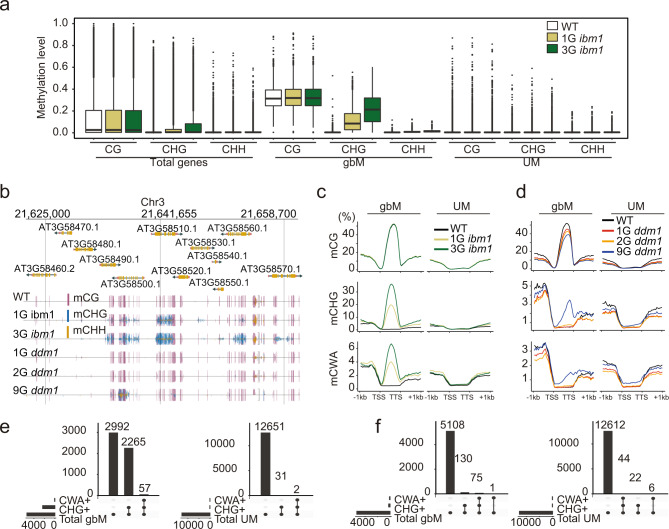
Ectopic CHG methylation of gbM genes increases over generations in *ibm1* and *ddm1* mutants. **a** The DNA methylation patterns of WT (Col-0), 1G ibm1, and 3G ibm1 mutants for all genes, gbM and UM genes (*N* = 33056, 5314 and 12684, biologically independent samples in each group of total, gbM and UM genes). Box plots show a median center line, the lower and upper hinges are the first and third quartiles. Whiskers represent 1.5x the interquartile range. **b** A genome browser view of genes with ectopic non-CG methylation in ibm1 and ddm1 mutants. **c** DNA methylation patterns of WT (Col-0), 1G ibm1 and 3G ibm1 mutants for gbM and UM genes. **d** mCHG-gain (>0.1) and mCWA-gain (>0.1) in 1G ibm1 and enriched in gbM genes. **e** DNA methylation patterns of WT (Col-0), 1G ddm1, 2G ddm1, and 9G ddm1 mutants for gbM and UM genes. **f** Gain of non-CG methylation in 9G ddm1 mutants is enriched in gbM genes. Source data underlying Figs. 1a, 1c, and 1d are provided as a Source Data file.

Phenotypic variation of certain mutants, namely *met1, ddm1*, and *ibm1*, becomes stronger over repeated generations of self-crossing^27,30,32,34^. To understand the possible reasons for this observation, previous studies examined the DNA methylomes of *ibm1* and *ddm1* separated by up to eight generations of single seed descent, respectively^30,40–42^. These analyses uncovered non-parental epialleles in the form of ectopic hypermethylation of CHG specifically in genes, which was expected for *ibm1*^43^, but was rather unexpected for *ddm1*. To explore the fate of these non-parental epialleles further, we measured CG, CHG and CHH methylation in *ibm1* mutants selfed for one and three generations using previously published data (Fig. 1a). We categorized genes into gene body DNA methylated genes (gbM, *N* = 5,314) and unmethylated genes (UM, *N* = 12,684) (Supplementary Data 3). Although it was known that ectopic CHG methylation occurs in genes, our analysis reveals that this ectopic methylation is essentially exclusive to gbM genes (Fig. 1a–d). Over 40% (2,265/5,314) of the gbM genes acquired ectopic CHG methylation in the first-generation mutant, which is highly statistically significant (Fisher’s exact test, *p*-value < 0.00001), compared to less than 1% (31/12,684) of the UM genes (Fisher’s exact test, *p*-value = 1, Fig. 1d; Supplementary Data 4). Furthermore, the levels of ectopic methylation increased over generations and was also found at CWA sites, which is catalyzed by CMT2 (Fig. 1b–d, Supplementary Figs. 1a, 2a, b). Together, these results are consistent with a proposed model whereby loss of IBM1 activity leads to ectopic H3K9me2 specifically in gene bodies of gbM genes, which recruits CMT2 and CMT3 to methylate CWA and CHG sites, respectively^43,44^. The specificity to gbM versus UM genes is most likely due to ectopic H3K9me2 that results from the activity of the histone lysine nine methyltransferases, SUV4/5/6, which are targeted to methylated DNA via their SRA domains^45^. The activity of this positive feedback loop specifically at gbM genes is consistent with the increase in DNA methylation and with the increased phenotypic variation of *ibm1* mutants over time.

The increase in phenotypic variation over generational time is much stronger in *ddm1* compared to *ibm1*, although the *ibm1* mutant used has not been propagated for as many generations as *ddm1*. Furthermore, much of the phenotypic variation in *ddm1* is mostly due to the loss of maintenance of DNA methylation over generational time. A similar analysis was performed for data from 9th generation selfed *ddm1*, which revealed a reduction of CG methylation in gbM genes (Fig. 1d) and showed ectopic CHG and CWA in genes (Fig. 1b, e, Supplementary Figs. 1b, 2c, d). However, although there was a greater probability of gbM genes acquiring ectopic methylation (2.5%, 130/5,314, Fisher’s exact test, *p*-value = 8.85e^−10^), it was much less than what is observed in *ibm1* and the ectopic methylation was also observed in UM genes albeit at a much lower frequency (0.17%, 22/12,684, Fisher’s exact test, *p*-value = 1) (Fig. 1f; Supplementary Data 4). Combined, these results show that loss of IBM1 activity immediately and directly affects ectopic methylation of gbM genes almost exclusively, whereas loss of DDM1 activity leads to preferential accumulation of non-parental epialleles via ectopic methylation with a preference for gbM genes compared to UM genes.

### Abundance of heterochromatin is associated with the frequency of spontaneous epialleles

DNA methylation feedback regulation between chromosomes is an emerging theme and has major implications for the formation of spontaneous epialleles^39^. One of the best examples of this process is nicely demonstrated using the *ddm1* epiRIL population that had variation in the number of chromosomal regions inherited from the *ddm1* parent versus the wild-type parent. It was observed that hypomethylated chromosomes from *ddm1* led to ectopic de novo methylation at regions inherited from wild type and that the amount of hypermethylation was negatively correlated with the abundance of heterochromatin^30^.

**Fig. 2:**
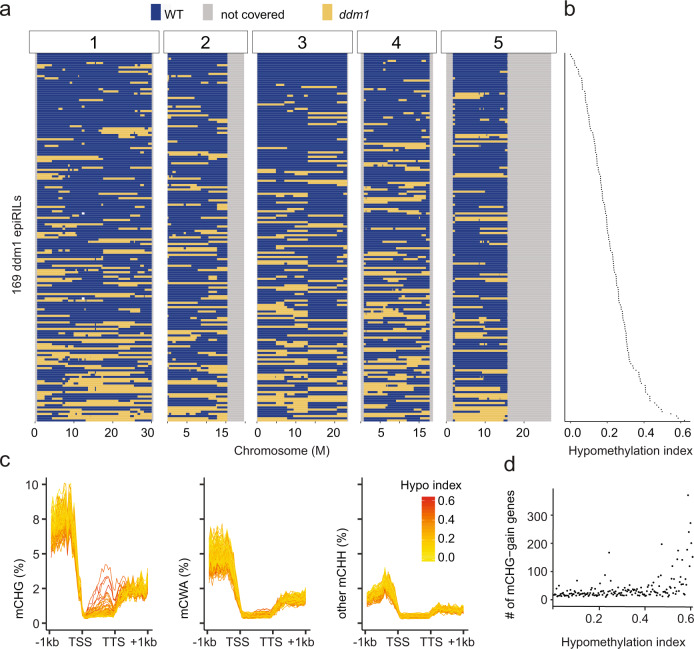
Hypomethylated chromosomes from the *ddm1* parent lead to ectopic DNA methylation in gbM genes. **a** A haplotype map for 169 *ddm1* epiRIL lines. Each row represents the genome composition of one epiRIL line which was ordered based on hypomethylation index which indicates amounts of DNA inherited from the *ddm1* parent. Yellow bars represent regions from *ddm1* parent, blue bars represent regions from wild-type parent and gray bars show regions not covered by markers. **b** Hypomethylation index is calculated as an average of the values on 144 DMR markers (methylated WT marker =0, unmethylated *ddm1* marker =1). So higher the value represents a more hypomethylated genome. **c** Metaplot shows non-CG methylation pattern on gbM genes for 169 *ddm1* epiRIL lines. Methylation patterns for each sample is shown by one line that was colored based on its hypomethylation index. **d** The scatter plot indicates the number of gbM genes that gain CHG methylation for each sample. Source data underlying Figs. 2a, 2c, and 2d are provided as a Source Data file.

The initial observations of the *ddm1* epiRIL population were examined using microarray technology^30^, which does not provide the opportunity to evaluate methylation in specific contexts. As a result, WGBS on a subset of three epiRILs was used to further reveal the type of hypermethylation present^30^. To further expand on these intriguing initial observations, we performed WGBS on 169 individuals from the *ddm1* epiRIL population (Supplementary Data 1) that are unique from the previously studied set of 123 lines using tiling arrays^46^. Using differentially methylated regions (Supplementary Data 5) we were able to create a haplotype map of each line that differentiated whether a chromosomal segment was inherited from wild type versus *ddm1* (Fig. 2a). Each *ddm1* epiRIL was assigned a hypomethylation index based on the amount of DNA inherited from the *ddm1* parent and the haplotype map (Fig. 2b). The higher the hypomethylation index indicates increased amounts of DNA from the *ddm1* parent is present in the line. Next, we examined ectopic DNA methylation in gbM genes **(**Supplementary Fig. 3**)** and observed an enrichment of ectopic CHG and to a certain extent CWA methylation, which was most apparent in lines with a very high hypomethylation index (Fig. 2c). Collectively, these data support that hypomethylated chromosomes from the *ddm1* parent led to in imbalance in DNA methylation patterns, resulting in ectopic activity of CMT2 and CMT3 at gbM genes

### Heterochromatin impacts spontaneous epiallele formation in trans

**Fig. 3:**
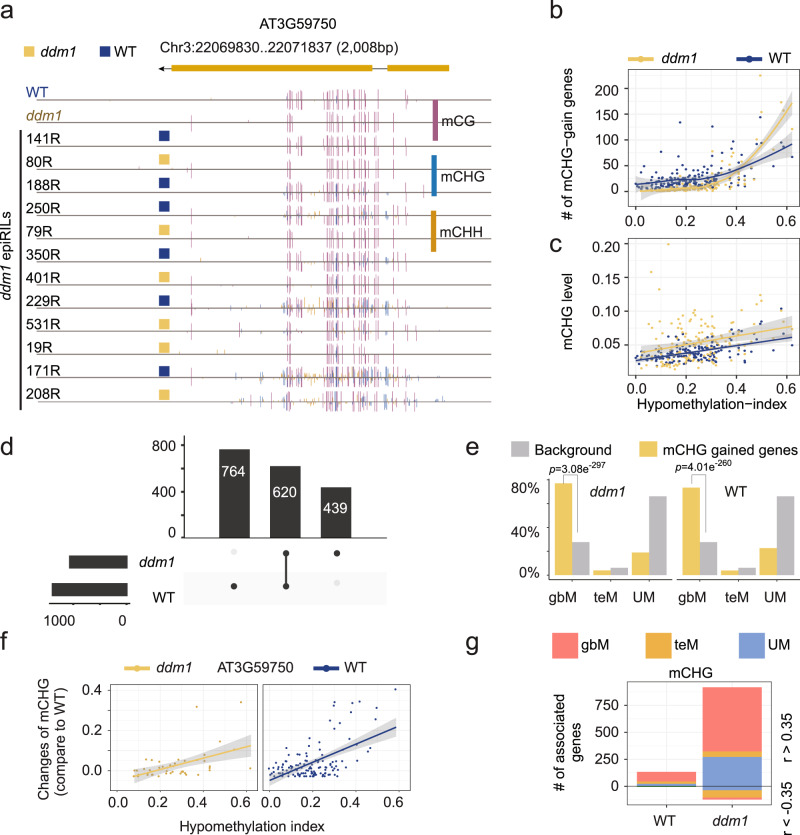
Global hypomethylation leads to ectopic activity of CMT3 at genes present within wild-type and *ddm1* haplotypes. **a** Genome browser view of a representative gene AT3G59750, in which non-CG methylation was observed in epiRILs with either wild-type or *ddm1* derived haplotypes. **b** Correlation between hypomethylated genome and the number of gbM genes that gain CHG methylation. Genes inherited from WT (blue dots) and *ddm1* (yellow dots) parents were calculated separately for each epiRIL. Gray area shows 95% confidence level interval for predictions (shown by lines) from a loess regression model. **c** Correlation between hypomethylated genomes and the average accumulated CHG methylation level on mCHG-gain genes. Gray area shows 95% confidence level interval for predictions (showing by lines) from a linear regression model. **d** A union set of total mCHG-gain genes from all epiRIL lines. It shows the number of mCHG-gain genes from each haplotype type as well as those shared by both haplotypes. **e** An enrichment analysis using Fisher’s Exact test shows that mCHG-gain genes that either from *ddm1* (left) or WT haplotypes (right) are enriched in gbM genes. Yellow bars show the ratio of each type of genes with ectopic mCHG. Gray bars show the ratio of each type of genes in comparison with all coding genes. *p-* value is based on one-sided test with alternative hypothesis that odds ratio is greater than 1. **f** Genome-wide association of methylation level changes (difference of value compared to WT) at an example gene compared to its hypomethylation index. An example of positive correlation is shown for AT3G59750. For each gene, the epiRILs were grouped based on haplotypes, either WT (blue) or *ddm1* (yellow) derived haplotypes. Gray area shows 95% confidence level interval for predictions (showing by lines) from a linear regression model. **g** Correlation test in f was applied for all genes. The bar plot shows the number of genes with CHG methylation level changes strongly correlated with the hypomethylation index (with Pearson correlation coefficient either >0.35 (above the line) or <−0.35 (below the line)). The number of associated genes is calculated separately based on haplotypes (either WT or *ddm1* derived haplotypes) and gene type (gbM, teM and UM). Source data underlying Figs. 3b, 3e, and 3g are provided as a Source Data file.

To further evaluate the impact to the disruption to normal DNA methylation states, we analyzed genes based on whether they were inherited from wild-type versus *ddm1* derived haplotypes. Ectopic activity of CMT3 and CHG methylation was observed at gbM genes (Fig. 3a) regardless of which haplotype they were present within and this ectopic activity correlated with the hypomethylation index (Fig. 3b, c; Supplementary Data 6). Importantly, this ectopic methylation was not due to disruption to maintenance of methylation within the 7^th^ intron of IBM1, which is known to lead to ectopic mCHG in genes (Supplementary Fig. 4). In total, ectopic CHG methylation was observed at 1,384 and 1,059 genes in wild-type and *ddm*1 haplotypes, respectively (Fig. 3d; Supplementary Data 7). A deeper inspection of these genes revealed a significant enrichment for ectopic activity specifically at gbM genes compared to teM genes and UM genes (which actually had a strong depletion compared to background expectations—Fig. 3e). Next, we evaluated genes on a case-by-case basis to determine how many of them were susceptible to feedback regulation of DNA methylation. For example, the gene presented in Fig. 3a shows a positive correlation between the change of CHG methylation and the hypomethylation index regardless of haplotype the gene resided within (Fig. 3f). Therefore, we performed a correlation analysis between the difference in CHG methylation between epiRILs and wild type for each gene that has evidence for ectopic CHG methylation in any line. These results revealed, yet again, a strong preference for gbM genes compared to UM and teM genes (Fig. 3g). Collectively, these results demonstrate that a greater amount of DNA hypomethylation, which is associated with a greater reduction to heterochromatin methylation, leads to ectopic activity of CMT3 in genes, with a clear preference for gbM loci.

### Heterochromatin is a hotspot for methylation QTL^epi^

**Fig. 4:**
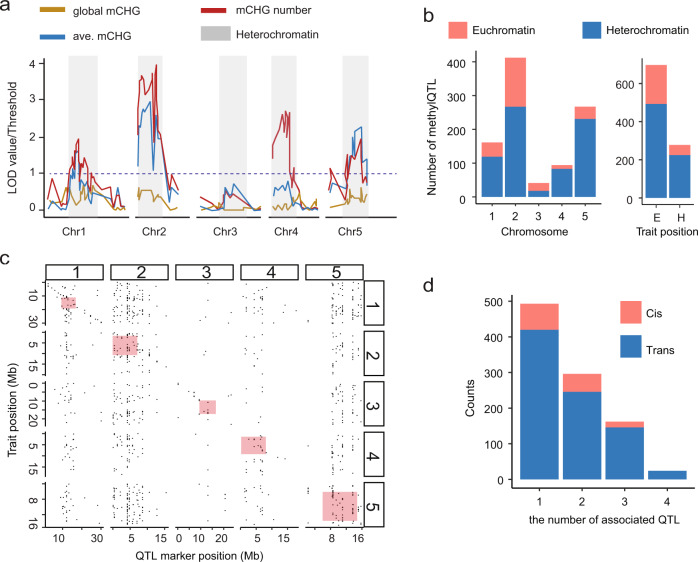
Methylation QTL for genes with ectopic mCHG are especially enriched in the pericentromeric regions. **a** Standard interval QTL mapping for genic mCHG related phenotypes. Methylation status (methylated, unmethylated) on 144 stably inherited DMR makers was used for QTL mapping. The results for three phenotypes, global mCHG level (global mCHG), the number of mCHG-gain genes (mCHG #), averaged mCHG level on genes (ave. mCHG), were plotted as likelihood-ratio test statistic (LOD score divided by threshold value of 3) against physical location of markers (Mb). **b** Barplots shows that methylQTL are especially enriched in the pericentromeric regions in comparison to euchromatin, whereas affected genes are enriched in euchromatin (right bar plot). E=Euchromatin and H=Heterochromatin. **c** The scatter plot shows the location of the 701 genes against the location of their corresponding methylQTL, respectively. **d** A comprehensive QTL analysis for each of the1595 genes that had evidence of ectoipc mCHG. Significantly associated QTL loci were identified for 701 genes. For association detected, half of them were linked with a single methylQTL, and others were found to be associated with 2–4 methylQTL. The majority of methylQTL are distant (trans) to associated genes. Source data underlying Fig. 4b–d are provided as a Source Data file.

The fact that genes in both wild-type and *ddm1* haplotypes are almost equally affected indicates that they are regulated in trans (by distant loci). To test this hypothesis, we used a methylation QTL^epi^ (meQTL^epi^) approach to explore the potential causal basis for the observed spontaneous hypermethylation in the *ddm1* epiRILs. Traditional genetic markers could not be used given the reduced genetic variations in these lines. Instead, we used differentially methylated regions (DMRs) that segregate in a stable, Mendelian fashion in this population (Supplementary Data 5), as markers for QTL analysis^9^. Using the single base resolution methylomes from 169 *ddm1* epiRILs we verified, improved and increased coverage of the existing map compared to previous attempts. We scanned each chromosome for associations with global mCHG, average mCHG of genes and the number of mCHG-gain genes within each epiRIL line (Fig. 4a; Supplementary Data 8 and 9**)**. There were no obvious associations with global levels of mCHG, however, the pericentromeric regions of multiple chromosomes were associated with the average mCHG levels of genes as well as the number of genes with ectopic mCHG (Fig. 4a**)**. To study this further, we performed a comprehensive QTL analysis for each of the 1,595 genes that had evidence of ectopic mCHG. In total, 701 of these genes were associated with meQTL^epi^ and the majority (718/975) of the QTL resided in heterochromatin regions (Fig. 4b). A genome-wide view of these events shows that distant (trans) meQTL^epi^ are especially enriched in the pericentromeric regions (Fig. 4b, c; Supplementary Data 10 and 11**)**. Curiously, chromosome 3 was somewhat devoid of meQTL^epi^ in contrast to other chromosomes, which was unexpected given the presence of well-known repeats such as a 5 kb chloroplast insertion, an rDNA and a telomeric repeat. Of the associations detected, most were linked with a single meQTL^epi^, although numerous loci that gained mCHG in the gene body were found to be associated with 2–3 meQTL^epi^ (Fig. 4d). Collectively, these data show that disruption to DNA methylation at heterochromatin that was triggered by the initial loss of *ddm1* leads to spontaneous epiallele formation at hundreds of loci across the chromosomes. Importantly, the ectopic CHG methylation is conditional on the heterochromatic state and does not segregate independently of it, indicating that there is constant feedback.

### H3K9me2 is important to the formation of spontaneous epialleles at unmethylated loci

The ectopic CHG and CWA methylation at gbM genes observed in *ibm1, ddm1* and *ddm1* epiRILs demonstrates independent paths for how positive feedback regulation between H3K9me2 and CMT2/CMT3 is established de novo. However, understanding the origins of spontaneous ectopic hypermethylation from these experiments is complicated by pre-existing DNA methylation at gbM genes. Previous research has shown that ectopic CHG accumulates in gene bodies in *met1*, which is devoid of CG methylation^11^. We re-evaluated *met1* WGBS data and found 1,161 genes that accumulate CHG methylation compared to wild type, with a strong statistical enrichment at gbM genes (Supplementary Fig. 5). However, loss of *MET1* leads to wide range of disruption to normal nuclear processes that could lead to indirect effects that explain these observations. Therefore, to understand how genes can spontaneously transition from unmethylated to methylated in a wild-type genotype, we took advantage of the *met1* epiRIL population^26^. In the *met1* epiRILs, large segments of chromosomes have lost gbM because they were derived from the *met1* parent where CG methylation is lost. Therefore, using these lines we can compare genes that maintain gbM versus those that were once gbM, but are now UM due to inheritance via *met1*.

**Fig. 5:**
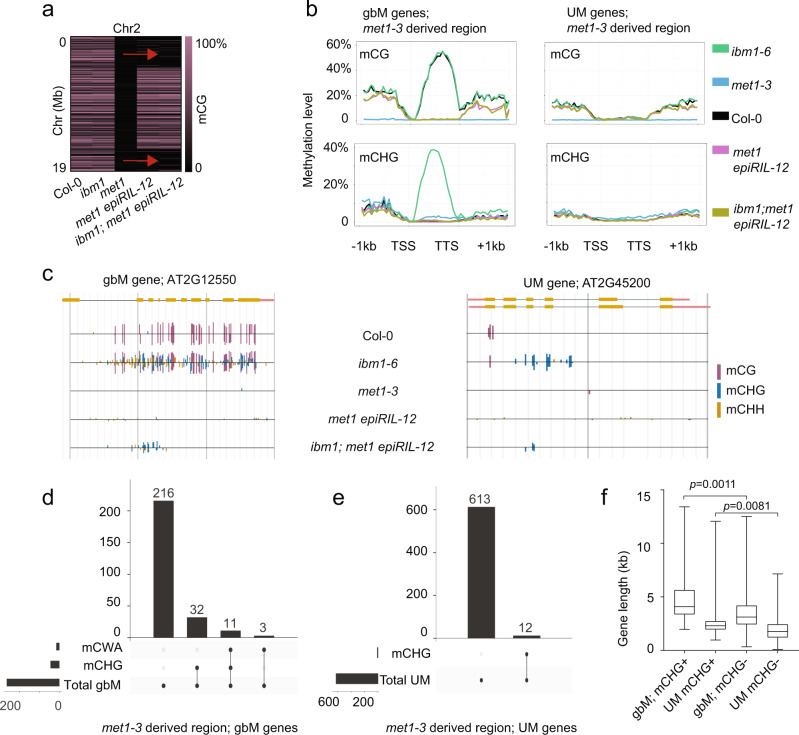
Mutations in *ibm1* lead to ectopic methylation preferentially in genes that previously possessed gbM. **a** A schematic showing regions that inherited mCG from the *met1 epiRIL-12* parent or the *ibm1-6* parent on Chromosome 2 (*Col-0, ibm1-6* and *met1-3* are shown as controls). Purple to black colors show the mCG percentage of genes that possess gbM in *Col-0* from 100% to 0%, respectively. Red arrows indicate a region in *ibm1; met1 epiRIL-12* derived from the original *met1-3* parent. **b** DNA methylation plot of gbM and UM genes including 1kb up and downstream for indicated genotypes and methylation contexts. Depicted regions shown are derived from *met1-3*. TSS: Transcription Start Site. TTS: Transcription Termination Site. **c** Genome browser examples of gbM and UM genes from *met1-3* derived region of the *met1 epiRIL-12* genotypes. **d** and **e** The number of gbM and UM genes that gained mCHG, mCWA or non-mCWA for in the *met1-3* derived region of *ibm1; met1 epiRIL-12*. Vertical bar plot shows count of overlapping genes between different methylation contexts. Horizontal bar plot shows count of specific type of genes. **f** Gene length distribution of genes that gained mCHG in gbM and UM genes derived from the *met1-3* region of *ibm1; met1 epiRIL-12*. N = 43, 12, 219, and 613 biologically independent samples for four groups from left to right. Gain is depicted by ‘+’ and no gain is depicted by ‘-’. *p*-value is generated by two-sided student *t*-test. Box plots show a median center line, the lower and upper hinges are the first and third quartiles. Whiskers represent 1.5x the interquartile range. Source data underlying Figs. 5b and 5f are provided as a Source Data file.

This design enables us to test the hypothesis that H3K9 methylation can establish itself de novo to recruit CMT2/3 activity to unmethylated genes. To increase the prevalence of H3K9me2, we crossed *ibm1-6* into the *met1* epiRIL-12 and isolated a homozygous *ibm1* line. We produced or used publicly available WGBS data from Col-0, *met1-3, ibm1-6, met1* epiRIL-12 and *ibm1;met1* epiRIL-12 (Supplementary Data 2^36,47^). Using CG methylation patterns, we were able to identify regions within each genotype that were derived from the *met1* versus the wild-type and/or *ibm1-6* parents (Fig. 5a and Supplementary Fig. 6). We identified 9.1 Mbs of sequence in *ibm1;met1* epiRIL-12 where 256 gbM genes had lost all CG methylation on chromosome 2 and were homozygous for *ibm1-6* (Fig. 5b). This *met1-*derived region also possessed 625 genes that were UM. We compared *ibm1-*induced ectopic DNA methylation of this collection of 881 genes, all of which have no CG methylation, yet have different histories of CG methylation (either gbM or UM - Fig. 5b). No obvious increase in ectopic CHG or CWA methylation was observed in *ibm1;met1* epiRIL-12 using a meta-analysis of these genes (Fig. 5b). Therefore, we performed a gene specific analysis, which identified a significant enrichment (46/256, Fisher’s exact test, *p*-value = 1.81e^−16^) of ectopic CHG and/or CWA methylation in genes that were at one time gbM compared to UM genes (12/625, Fisher’s exact test, *p*-value = 1) (Fig. 5c–e; Supplementary Data 12). The genes that acquired CHG methylation were on average much longer than genes that did not regardless of their historical gbM versus UM status (Fig. 5f). In summary, these results show that spontaneous epialleles can form in unmethylated genes and that there is a preference for genes that were gbM in previous generations. The reason for this preference is unknown, but it is likely in part due to the length and transcriptional activity known to be associated with gbM genes.

### Hotspots for epiallele formation have increased chromatin contacts with H3K9me2 regions

**Fig. 6:**
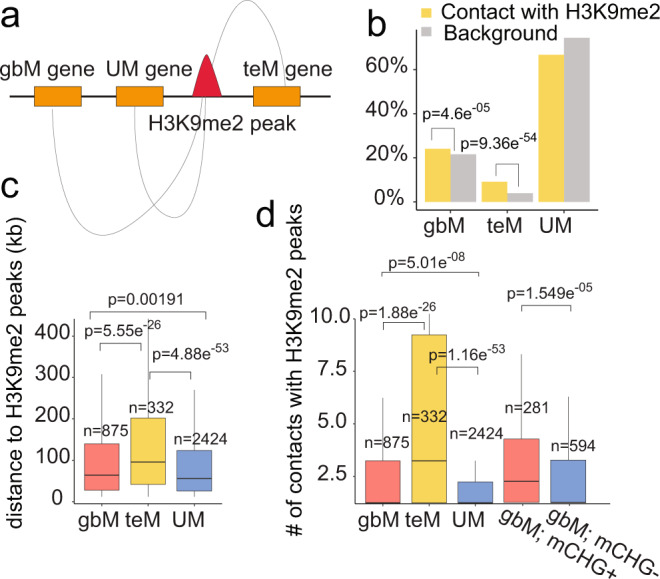
Loci that are susceptible to spontaneous epiallele formation have a greater contact frequency with H3K9me2 region. **a** Hi-C data was used for identifying significant three-dimensional contacts between genomic regions. The schematic plot depicts the interaction from regions possessing H3K9me2 and genes. **b** Enrichment test for genes that have contacts with H3K9me2. Yellow bars show the proportion of each type of genes that contacts with H3K9me2. Grey bars show the proportion of each type genes in comparison with all coding genes. Fisher’s exact test was used for enrichment test. *p*-value is based on one-sided test with alternative hypothesis that odds ratio is greater than 1. **c** Distribution of distance between three types of genes (gbM, UM, teM) and H3K9me2 regions. Two-sided Wilcoxon tests was used for pairwise comparison of each two groups without adjustment for multiple comparisons. **d** Distribution of the number of contacts between three types of genes (gbM, UM, teM) and H3K9me2 regions. gbM genes were further classified into a group with ectopic mCHG (gbM; mCHG+) and a group without ectopic mCHG (gbM; mCHG-) based on ddm1 mutant line data. Two-sided Wilcoxon tests was used for pairwise comparison of each two groups without adjustment for multiple comparisons. Box plots show a median center line, the lower and upper hinges are the first and third quartiles. Whiskers represent 1.5x the interquartile range. Source data underlying Fig. 6b–d are provided as a Source Data file.

Why certain genes are hotspots for epiallele formation versus others is unknown. One possible explanation for the preferential accumulation of mCHG in certain genes is that they are in nuclear neighborhoods/compartments that have higher concentrations of nucleosomes that possess H3K9me2. This increased localized pool of H3K9me2 could lead to spontaneous and rare incorrect incorporation of these nucleosomes into gene bodies when chromatin is reassembled after DNA replication. Once present in a gene body, CMT3 would bind H3K9me2 to methylate associated DNA^18,48^. Presumably, H3K9me2 would be removed by IBM1 once the gene is transcribed, but mCHG would still be present. To test the hypothesis, we used Hi-C data^49^, which incorporates proximity ligation, to reveal chromatin interactions. We identified all regions that have at least one edge of the chromatin interaction that overlaps with H3K9me2 (Fig. 6a). We discovered that gbM and teM genes are enriched, albeit weakly, for contacts with H3K9me2, whereas UM genes were not enriched (Fig. 6b). The distance between H3K9me2 regions that contacted gbM and UM genes were not significantly different from one another, yet the teM genes were significantly further away (Fig. 6c). What did distinguish gbM genes that were hotspots for spontaneous epiallele formation (ectopic mCHG) was that they had a greater contact frequency with H3K9me2 regions compared to UM genes (Fig. 6d). This result was further confirmed using Hi-C data from *ddm1*^50^. Although global hypomethylation in *ddm1* leads to a significant reduction of contact frequency (most obvious for teM genes) compared to wild-type Col-0, gbM genes still had a greater contact frequency with H3K9me2 regions compared to UM genes. Even though ectopic non-CG methylation is rare in the first generation of *ddm1* being a mutant, genes with ectopic mCHG did show a greater contact frequency with H3K9me2 regions (Supplementary Fig. 8). These results show that one possible mechanism by which certain loci are susceptible for spontaneous epiallele formation could be through association with H3K9me2 regions of the genome in three-dimensional space.

## Discussion

One possible reason for the prevalence of epialleles in plants is that there is no comprehensive erasure of DNA methylation from one generation to the next like there is in mammalian genomes^27,51–53^. Instead, extensive reinforcement of DNA methylation occurs during gamete production^54^, with the exception of rare events important for genomic imprinting. This feature of flowering plants enables the occurrence of rare spontaneous epialleles in a single cell to propagate in subsequent cell divisions and in some cases to become a major allele in the next generation if it is present in a cell that leads to the production of gametes. Therefore, once plant epialleles form, they can be studied to understand their stability over generations, interaction with alleles with different epigenomic states and association with phenotypes.

Another likely explanation that is consistent with the epigenetic nature of epialleles is the involvement of positive feedback loops. These positive feedback loops are fundamental to maintenance of DNA methylation in plants. Hemimethylated CGs are recognized by VARIANT IN METHYLATION 1^55^, which recruits MET1 to maintain CG methylation. CMT2 and CMT3 are directed to methylate DNA through their binding to regions with H3K9me2 and H3K9 methyltransferases bind methylated DNA and methylate H3K9^18–20,48,56–58^. Similarly, the H3K9 methyl binding protein SAWADEE HOMEDOMAIN HOMOLG1^59^ and the DNA methylation readers SUVH2/9 (Su(var)3-9 homolog) recruit the RdDM pathway to target sequences for DNA methylation reinforcement^45^. There are multiple examples in the literature that show the effectiveness of these feedback loops at re-establishing patterns of DNA methylation^23^. For example, multiple proteins have recently been used as triggers in epigenome editing whereby the protein is targeted to the FWA promoter to establish a DNA methylation^45,60^. Once a positive feedback loop is established the transgene triggers can be segregated away as they are no longer needed for maintenance^45^. Another recent study illustrated how RNAi-independent pathway(s) effectively re-establishes non-CG methylation at transposons that had lost all non-CG methylation due to loss of H3K9me2 and CMT2/3 activity^61^. Because CG methylation was still present at these transposons, it functioned to recruit SUV4/5/6 to re-establish H3K9me2 and non-CG methylation. It is well known how these pathways maintain DNA methylation, but how feedback regulation is established de novo at new regions in the genome is challenging to study given their spontaneous nature.

One possibility that is consistent with the spontaneous nature of epialleles is that the feedback regulation important to maintenance of DNA methylation have a low rate of off-targeting activity^57^. Although their main function is to target repeats and transposons for silencing, biochemical features of many of these components, such as domains that recognize methylated DNA (SRA, MBD) and histones (CHROMO, BAH, SAWADEE) leads to improper establishment of these pathways at unintended regions in the genome^57^. Given the function of IBM1, a histone demethylase that removes H3K9me2 from PolII-dependent transcribed regions of the genome, it seems likely that plant epigenomes have evolved mechanisms to reduce off-targeting activity^32,43,44,62^. In this particular case, pre-existing CG methylation at gbM genes serves as a substrate for the SRA domains in SUVH4/5/6 to catalyze H3K9me2^58^, which is counteracted by the activities of IBM1.

Our analysis of the *ibm1;met1 epiRIL-12*, shows that the absence of IBM1 does result in ectopic CMT3 activity at completely unmethylated genes, which suggests that H3K9me2 is likely a trigger for inducing spontaneous epialleles. Evidence that H3K9me2 can seed de novo methylation independent from RdDM and pre-existing DNA methylation has also been observed at the *BONSAI* locus in *A. thaliana*^42^. Therefore, understanding H3K9me2 dynamics and why certain genes are hotspots for H3K9me2 activity compared to others will be essential to understanding the origin of epialleles. The ectopic hypermethylated epialleles identified in the genotypes used in this study show that there is a non-random process that makes certain loci more susceptible to spontaneous epiallele formation (Supplementary Fig. 7). This has led us to speculate that gbM genes in angiosperms arise from off-targeting activity of positive feedback loops established between CMT3-H3K9me2 and that gbM is evolutionarily neutral. These genes are susceptible to this pathway because of their inherent functions. These genes are generally ‘housekeeping’ genes that are long, moderately expressed in all cells and typically not regulated by development/environment. Transcription at these loci is likely a prerequisite to becoming a gbM gene, transcription could lead to incorporation of H3K9me2 nucleosomes at a low rate. Typically, this H3K9me2 is removed by IBM1, but over evolutionary time mistakes occur which lead to ectopic activity of CMT3 leading to CHG methylation and eventually CG methylation. How CHG methylation transitions to CG methylation to provide transgenerationally stability of epialleles is currently unknown and was not addressed in this study. There are at least two possible mechanisms by which this occurs^63^. One includes the activity of the RdDM pathway, which is recruited to regions possessing H3K9me2 via SHH enabling DRM2 to methylate CGs. This could be especially prevalent during CHH DNA methylation reinforcement that occurs in the embryo and after fertilization^52,54,64–67^. The second possibility includes recruitment of VIM1 to methylated DNA via its SRA domain (in this case CHG methylation)^68^, which could lead to rare de novo methylation of CGs by MET1. Regardless of the exact mechanism of transitioning CHG to CG methylation, the incorporation of these feedback loops would be prevented at many developmental/environmental regulated genes as they are targeted by the Polycomb Repression Complex and H3K27me3 to precisely limit their expression^69^. H3K27me3 is incompatible with maintenance of DNA methylation in *A. thaliana*, as it colocalizes with H2A.Z^70^. Given H3K9me2 associates with H2A.W^71^, the presence of H2A.Z would prevent the CMT3-H3K9me2 feedback loop from establishing gbM at many genes in the genome.

We hypothesize that spontaneous epialleles form as a byproduct of enzymatic activities that are dedicated to the maintenance of heterochromatin and that variation in heterochromatin abundance and methylation influences the rate of spontaneous epiallele formation^35^. If genes that maintain heterochromatin sometimes act on inappropriate targets, causing epialleles, natural selection cannot reduce epialleles by reducing DNA methylation because that would lead to loss of maintenance of heterochromatin and result in genome instability. In this way, the evolution of epialleles is similar to the evolution of chromosome rearrangements. In that case, molecular recombination, is favored to generate gamete diversity (or for some other reason) that has an unintended, deleterious consequences, namely the production of genome rearrangements by ectopic recombination.

Numerous major questions remain, but the most important one is how is H3K9me2 initially incorporated into an unmethylated region de novo. Transcription coupled incorporation of H3K9me2 nucleosomes is one possibility given the role of nucleosome eviction and reincorporation during transcription^72^. It’s also possible that H3K9me2 is mis-incorporated to certain regions of the genome during the establishment of chromatin upon DNA replication. In this study, we used Hi-C data to show the spontaneous epialleles we identified interact with H3K9me2 regions at greater frequencies that unmethylated genes in the genome. This result supports that the hypothesis that there are sub-nuclear compartments that could have different concentrations of H3K9me2 nucleosome pools and that spontaneous epialleles are more likely to occur in pools with high concentrations of H3K9me2 nucleosomes. However, the current evidence to support these conclusions are premature to make stronger conclusions. Regardless, the involvement of CMT3-H3K9me2 feedback regulation is a major factor in the origins of spontaneous epialleles and future studies will be required to test these proposed models.

## Methods

### Plant material

The only new plant material generated for this study was the create of *ibm1;met1 epiRIL-12*. These individuals were produced by crossing *ibm1-6* (SALK_006042)^73^ to a *met1 epiRIL-12*^26^ and isolating homozygous *ibm1-6* lines in the F2. The complete set of *ddm1*-epiRILs from Johannes *et al*. was obtained from the Versailles Arabidopsis Stock center of INRA (http://publiclines.versailles.inra.fr/)^25^. All epiRIL lines were propagated in a greenhouse at the Leibniz Institute of Plant Genetics and Crop Plant Research (IPK). The plants were grown in single seed pots and at a later developmental stage six siliques per plant were left to dry. Seeds for a selection of 169 epiRILs were sown in the IPK fully automatic phenotyping facility for small plants (Junker et al., 2015). Three independent experiments in three consecutively months were performed. In each of the 3 cultivation experiments, 6 individual plants from each line were grown in 2 separate trays completely randomized in the chamber. The sown pots were firstly placed for 3 days at 4 °C in darkness and then the plants were acclimated for 2 days under 16/14 °C with reduced light conditions. Following this, they were grown under long day conditions (16h light, 8h dark) at 20/18 °C, 60–75% humidity and 180-240 μΕ light intensity. The pots were watered with 55, 30 and 20 mL water the 2, 7, and 8 day after sowing (DAS) and then every other day with 55 mL.

### Library construction

All epiRIL lines were harvested at 27 DAS in a time frame of 3 h. Flowering stems and roots were removed and all 18 individual plants from each epiRIL line were pooled in 50 mL tubes. They were immediately frozen in liquid nitrogen and stored to −80 °C until processing. Genomic DNA was extracted from each pooled sample using the DNAeasy plant mini kit from Qiagen. 169 epiRIL lines with at least 1 μg of DNA were submitted to the Beijing Genome Institute where they were prepared for WGBS libraries. Sequencing was performed on an Illumina HiSeq X ten instrument. Clean raw paired-end files were obtained from BGI and used for downstream analysis. The *ibm1;met1 epiRIL-12* library was prepared following the MethylC-seq protocol^74^. Briefly, genomic DNA was sonicated to 200 bp using a Covaris S-series focused ultrasonicator, and end-repaired using End-It DNA end-repair kit (Epicentre). End-repaired DNA was subjected to A-tailing using Klenow 3′–5′ exo− (NEB) and ligated to methylated adapters using T4 DNA ligase (NEB). Ligated DNA was subsequently bisulfite converted using the EZ DNA methylation-Gold kit as per the manufacturer’s instructions and amplified using KAPA HiFi uracil + Readymix Polymerase.

### Methylome mapping

The WGBS data from the *ddm1-*epiRILs was analyzed using the Methylstar v1.4 pipeline^75^. Region-level methylation calls were obtained with Methimpute v1.16.0 (200 bp bins, step size = 50 bps, at least 10 cytosines per bin)^76^. We used Methimpute’s 2-state Hidden Markov Model to classify a given region as either homozygous methylated or homozygous unmethylated. These state calls were used downstream for the construction of an augmented linkage map in the epiRIL panel. Base-resolution methylome analysis of the *ddm1*-epiRILs, *ibm1* and *met1* epiRIL12 data used in this study were all processed by Methylpy v1.3 as described in^77^. Quality filtering and adapter trimming were performed using cutadapt v1.9.dev1^78^. Qualified reads were aligned to the *A. thaliana* TAIR10 reference genome^79^ (downloaded from https://phytozome.jgi.doe.gov) using bowtie 2.2.4^80^. Only uniquely aligned and nonclonal reads were retained. Chloroplast DNA (which is fully unmethylated) was used as a control to calculate the sodium bisulfite reaction non-conversion rate of unmodified cytosines. A binomial test was used to determine the methylation status of cytosines with a minimum coverage of three reads.

### Data acquisition

WGBS data of *ddm1* and *ibm1* mutants and their self-crossing offspring used in this analysis were obtained from published datasets^30^. WGBS data of *met1-3*, *ibm1-6* and *met1* epiRIL-12 were obtained from previously published datasets^36,47^. Hi-C data and H3K9me2 ChIP-seq reads used in this study were obtained from a previously published datasets^49,81^.

### Gene body methylation status classification

To explore the fate of ectopic methylation of genes that have different DNA methylation states, we categorized genes into one of three classes including gbM, teM and UM based on CG, CHG and CHH methylation in wild-type Col-0. The total number of cytosines and the methylated cytosines were counted for cytosines in each context (CG, CHG, and CHH) for the coding sequences (CDS) of the primary transcript for each gene. The percentage of methylated sites for each sequence context in all coding regions were used as the background probability of having methylation on a single site. Given a background probability and the total number of cytosines and methylated cytosines, a *p*-value was calculated using a binomial distribution to show the cumulative probability of having a higher number of methylated cytosines on a given gene^82^. Then a *q*-value was calculated by adjusting *p-* values by Benjamin–Hochberg FDR to control the false discovery rate.

Genes were classified as gbM if they had reads mapping to at least 20 CG sites and had a *q*-value < 0.05 for mCG and a *q*-value > 0.05 for mCHG and mCHH. Genes were classified as mCHG if they had reads mapping to at least 20 CHGs, a mCHG *q*-value < 0.05, and a mCHH *q*-value > 0.05. As mCG is commonly associated with mCHG, the *q*-value for mCG could be significant or insignificant in mCHG genes. Genes were classified as mCHH if they had reads mapping to at least 20 mCHH sites and a mCHH *q*-value < 0.05. *q-v*alues for mCG and mCHG could be anything as both types of methylation are associated with mCHH. mCHG and mCHH genes were collectively referred to as teM genes. Genes were classified as unmethylated (UM) if they had reads mapping to at least 20 mCHH sites and had a *q*-value > 0.05 for all sequence contexts. To make sure the selected UM genes are truly unmethylated, we further remove genes with more than 2 symmetric mCG from UM genes list. Only genes that fit the definition of gbM, teM and UM above were used for the downstream analysis in this study.

### Determination of DNA methylation patterns at genes

For *ibm1*, *ddm1* and *ddm1* epiRIL lines, DNA methylation patterns at gbM and UM genes were explored. Each gene was divided into 20 windows. Additionally, regions 1000 bp upstream and downstream were each divided into 20 50-bp windows. Methylation levels (total methylated reads divided by total reads mapped to cytosine sites in a window) were calculated for each window^77^, and a mean value for each window was averaged over the methylation level of the same window from genes in the same group (gbM/UM). The mean methylation levels and their corresponding window number was used to generated metaplots using the R package ggplot2.

### Locating the met1-derived regions in *ibm1;met1 epiRIL-12*

The *met1 epiRIL-12* line used in this study was carefully selected to ensure the *IBM1* locus was derived from the Col-0 parent instead of the *met1* parent. The *met1 epiRIL-12* derived regions of *ibm1;met1 epiRIL-12* was identified by comparing CG methylation levels on the exons of gbM genes between the epiRIL line and its parents (*ibm1-6* and *met1 epiRIL-12*). Each chromosome sequence was divided into continuous bins and each bin included at least 10 exons. A bin with an average mCG level that was greater than 25% reduced in *ibm1;met1 epiRIL-12*, as compared to that in *ibm1-6* was used to differentiated the *ibm1-6* and *met1 epiRIL-12* derived regions. All downstream analyses of this line used the same criteria described in ‘Gene body methylation status classification’ section of the Methods for defining gbM and UM genes. Regions that possessed *met1 epiRIL-12* derived regions in the *ibm1-6* mutant background were used to evaluate the consequence of mutant *ibm1-6* on genes that had lost gbM due to previous loss of *met1*. Ectopic CHG or CWA methylation on gbM and UM genes required a minimum of three sites with an average 10% higher methylation level when compared to the wild-type Col-0 parent.

### Construction of an augmented epiRIL linkage map

Previously, we used tiling-array data from 123 epiRILs and their *ddm1-2* and Col-wt founder lines and identified differentially methylated regions (DMRs) in the founders that were stably inherited in the epiRILs through at least 10 rounds of meiosis^46^. Using these ultra-stable DMRs as physical markers we were able to construct a linkage map involving 126 DMRs in this isogenic experimental system^46^, and later used this map for QTL^epi^ analysis^9^. The 169 epiRIL methylomes from the present study were employed to augment the existing linkage map, with the goal to improve marker spacing and mapping resolution. Among the 169 epiRILs measured in the present work, only 37 overlapped with the 123 epiRILs used in the previous our studies^9,46^. We used the tiling-array-derived linkage map of 126 DMRs as a starting point. For the 37 overlapping epiRILs, the WGBS derived methylation state calls at these 126 DMR position were consistent with the previous tiling-array calls, indicating that these DMRs are robust. For a given chromosome, we stepped in new DMRs into the existing linkage map. DMRs that correlated significantly across chromosomes with any of the core DMRs were rejected. For map cleaning we employed Rqtl’s tutorial on map construction^83^. For DMRs with identical cM positions we selected those that best optimized overall marker spacing in terms of base pair coverage. DMRs were kept or rejected by manual inspection in conjunction with the likelihood score from the drop.one function in Rqtl. The final linkage map contains 144 well-spaced markers, which provide improved coverage in chromosome arms.

### Methylation analysis for *ddm1* epiRILs

For methylation analysis in *ddm1* epiRILs, we needed to determine whether a chromosomal segment was inherited from the wild-type or *ddm1* parent for each line. The methylation levels at 144 selected DMR markers was calculated for each line. DMRs were classified as an unmethylated *ddm1* marker if their methylation level was less than 0.5. Otherwise, DMRs were classified as methylated WT makers if their methylation level was greater or equal to 0.5. Then, the haplotype map in Fig. 2a was generated based on the location of 144 DMRs and their methylation status (methylated, unmethylated) for each line using the R package ggplot2. We also used a measurement of hypomethylation index to determine the amount of DNA inherited from the *ddm1* parent. The hypomethylation index was calculated as an average of the values at 144 DMR markers (methylated WT marker =0, unmethylated *ddm1* marker =1). A higher value represents a more hypomethylated genome. To further evaluate the impact to the disruption to normal DNA methylation states, we classified genes into wild-type versus *ddm1* parent-derived regions based on the haplotype map for each line. Then, we explored how ectopic CHG methylation was distributed at genes from WT versus *ddm1* parents, respectively. A Fisher’s Exact test was used to evaluate the enrichment of mCHG-gain genes in three categories of genes (gbM, UM, teM). A Pearson correlation coefficient was used to evaluate how CHG methylation changes against hypomethylation index for each gene.

### QTL mapping analysis

Using the binary methylation status (M/U) at 144 DMR markers of each epiRIL line as genotypic data together with the genic mCHG related phenotypes, including three global genic mCHG related traits and genic mCHG levels on each of the 1595 mCHG-gain genes, we performed interval mapping with Rqtl. The customized R scripts used for the QTL mapping analysis can be obtained from the GitHub repository provided in the Code Availability section^9^.

### Characterizing chromatin interactions between genes and H3K9me2

We used previously published Hi-C data^49^ to identify chromatin contacts between two genomic regions in nonadjacent locations along the genome. The HiC-Pro v2.11.4 pipeline was used to process Hi-C data, from raw reads to normalized contact matrices for selected windows using the parameter ‘–binsize 2000’, since 2 kb is an approximate value of the average gene length of *A. thaliana*^84^. The contact matrices were then transformed to Fit-Hi-C v.2.0.7 readable input files with hicpro2fithic.py. Then, significant contacts were identified using Fit-Hi-C by assessing the enrichment of observed from the expected contact counts^85^. Raw reads of H3 and H3K9me2 ChIP-seq were trimmed with Trim Galore v0.6.5 (https://github.com/FelixKrueger/TrimGalore) with default parameters. The remaining reads were aligned to the *A. thaliana* TAIR10 reference genome using Bowtie2 v2.3.5.1 with default setting. Aligned reads were sorted using SAMtools v1.10^86^. Then, unmapped reads, duplicated reads and multiple mapped reads were filtered out using Sambamb v0.7.1^87^. Lastly, the remaining uniquely mapped reads were used in MACS2 for peak calling with parameters ‘-g 1.35e+8–broad’^88^. H3 ChIP-seq was used as a control for identification of H3K9me2 enriched regions. Significant contacts that had at least one edge that overlaps with a H3K9me2 enriched region and another edge that overlapped with a gbM (*n*=875), teM (*n*=332) or UM (*n*=2424) gene was selected for downstream analysis. A Fisher’s Exact test was used to evaluate the enrichment of genes that have contacts with H3K9me2 peaks in the three categories of genes (gbM, UM, teM). The distribution of distance and the number of contacts between three categories of genes and H3K9me2 peaks were compared using a Two-sample Wilcoxon test. The selected gbM genes (n=875) were further classified into an ectopic mCHG-gain group (gbM; mCHG+) and a group without ectopic mCHG (gbM; mCHG-) based on the methylome of *ddm1* mutant. The distribution of the number of contacts between these two groups and H3K9me2 peaks were also compared using a Two-sample Wilcoxon test.

We also used previously published Hi-C data to identify chromatin contacts between two genomic regions in nonadjacent locations along the genome for both wild-type Col-0 and *ddm1* mutant^50^. Significant contacts were obtained from Hi-C data using HiC-Pro and Fit-Hi-C following the same pipeline and parameters as the previous analysis (see the first paragraph in ‘Characterizing chromatin interactions between genes and H3K9me2’ section of the Methods) in Fig. 6. Significant contacts that had at least one edge that overlaps with a H3K9me2 enriched region and another edge that overlapped with a gbM, teM or UM gene were selected. The number of the three types of genes (gbM/teM/UM) used in the analysis were shown separately for both wild-type and *ddm1* mutant samples in Supplementary Fig. 8. The gbM genes (*n*=1775) selected from *ddm1* Hi-C data were further classified into an ectopic mCHG-gain group (mCHG+) and a group without ectopic mCHG (mCHG-) based on methylome of *ddm1* 1G mutant. The distribution of the number of contacts between these two groups and H3K9me2 peaks were compared by Two-sample Wilcoxon tests.

### Box plots

All box plots presented show a median center line with an upper and lower quartile. Whiskers represent 1.5x the interquartile range.

### Reporting summary

Further information on research design is available in the Nature Research Reporting Summary linked to this article.

## Supplementary information

Supplementary Information

Description of Additional Supplementary Files

Supplementary Data 1

Supplementary Data 2

Supplementary Data 3

Supplementary Data 4

Supplementary Data 5

Supplementary Data 6

Supplementary Data 7

Supplementary Data 8

Supplementary Data 9

Supplementary Data 10

Supplementary Data 11

Supplementary Data 12

Reporting Summary

## Data Availability

Previously published sequencing data used in this study are available listed in Supplementary Data 2. WGBS sequencing data produced from this study have been deposited in the NCBI GEO database under GSE171157 and GSE171414. Source data are provided with this paper.

## Source data

Source Data

## Code availability

Code used in processing and analysis of these data can be found at GitHub [https://github.com/schmitzlab/Heterochromatin-quantitative-epiallele-formation]^89^.
