# Supplementary material for: Heterochromatin is a quantitative trait associated with spontaneous epiallele formation
Source: Nat Commun. 2021 Nov 29;12:6958. doi: 10.1038/s41467-021-27320-6 (PMC8630088; doi:10.1038/s41467-021-27320-6)
Supplement: Supplementary file 1 — Supplementary Information [file 41467_2021_27320_MOESM1_ESM.pdf]

Heterochromatin is a quantitative trait associated with spontaneous epiallele formation

Zhang *et al*

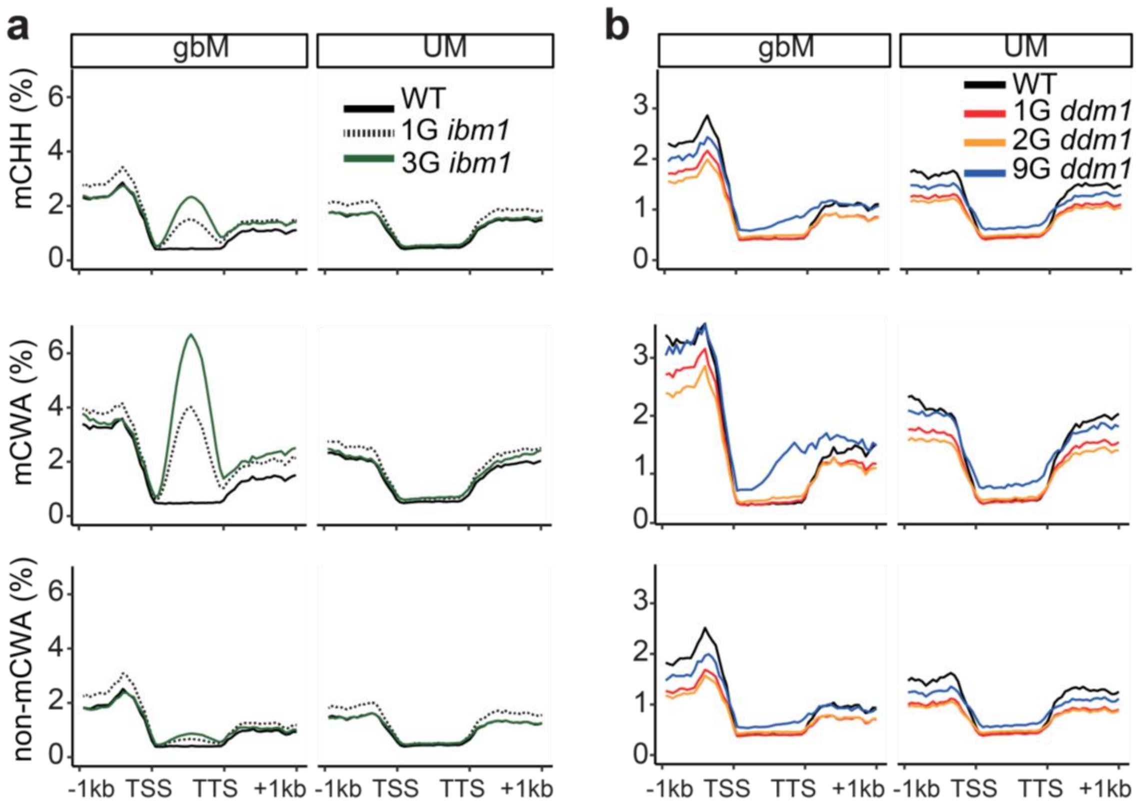

Supplementary Figure 1. Ectopic CHH methylation on genes are mainly at CWA sites in *ibm1* and *ddm1* mutants. a CHH methylation of WT (Col-0), 1G *ibm1*, and 3G *ibm1* mutants for gbM and UM genes.**b** CHH methylation of WT (Col-0), 1G *ddm1*, 2G *ddm1*, and 9G *ddm1* mutants for gbM and UM genes. Source data are provided as a Source Data file**.**

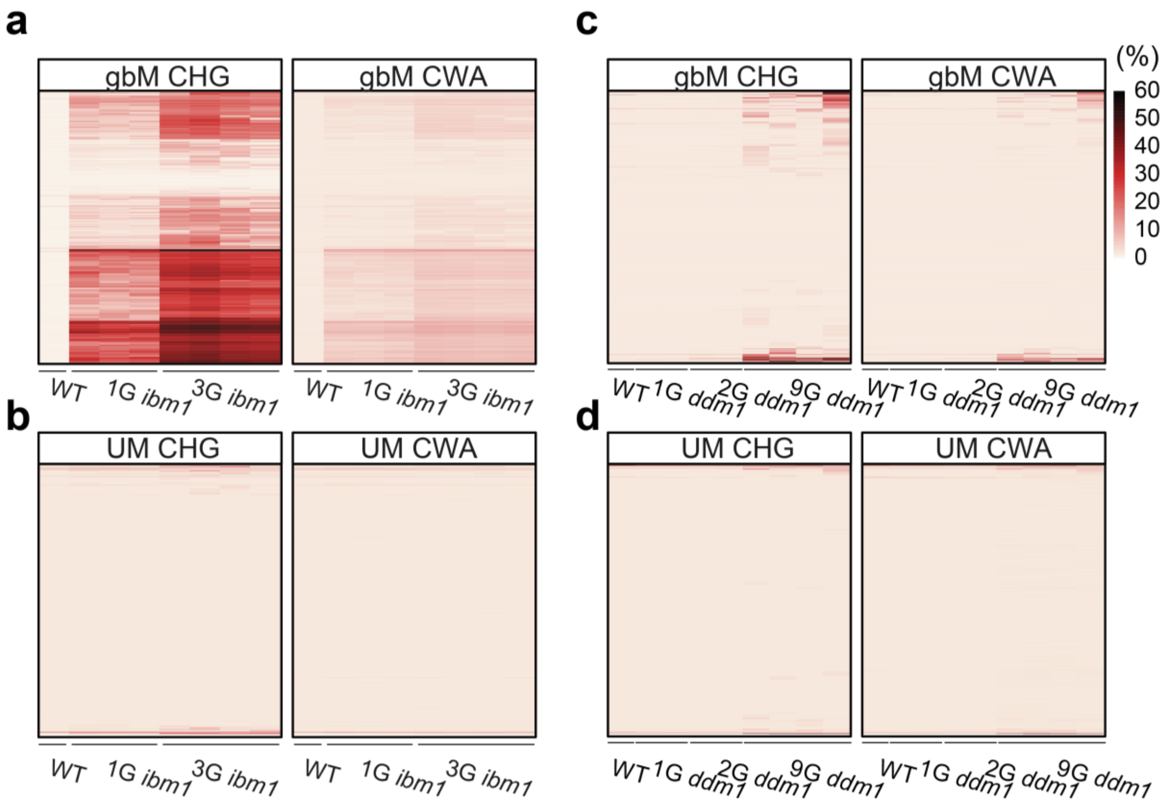

Supplementary Figure 2. Gradual increase of non-CG methylation at gbM genes in *ibm1* and *ddm1* mutants over generations. a Heatmaps show mCHG and mCWA increasing over generations on gbM genes in *ibm1* mutants. **b** mCHG and mCWA patterns on UM genes in *ibm1* mutants. **c** mCHG and mCWA increasing over generations on gbM genes in *ddm1* mutants. **d** mCHG and mCWA on UM genes in *ddm1* mutants. Source data are provided as a Source Data file**.**

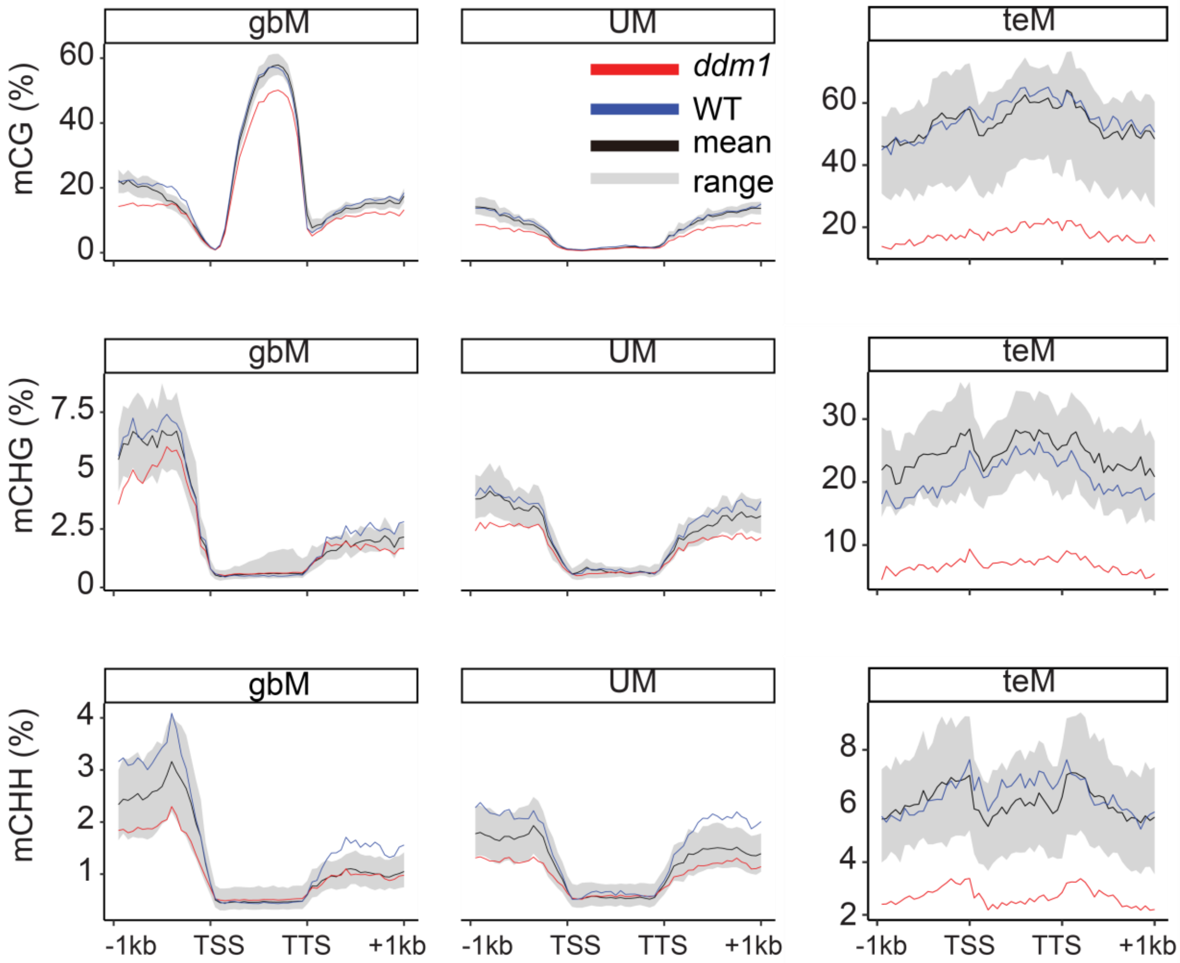

Supplementary Figure 3. DNA methylation patterns at gbM, UM and teM genes for *ddm1* epiRILs. Red and blue lines show the methylation pattern for *ddm1* and WT parents. Black line shows the averaged methylation level of 169 epiRILs. Grey bands show the maximum and minimum value of those epiRILs. Source data are provided as a Source Data file**.**

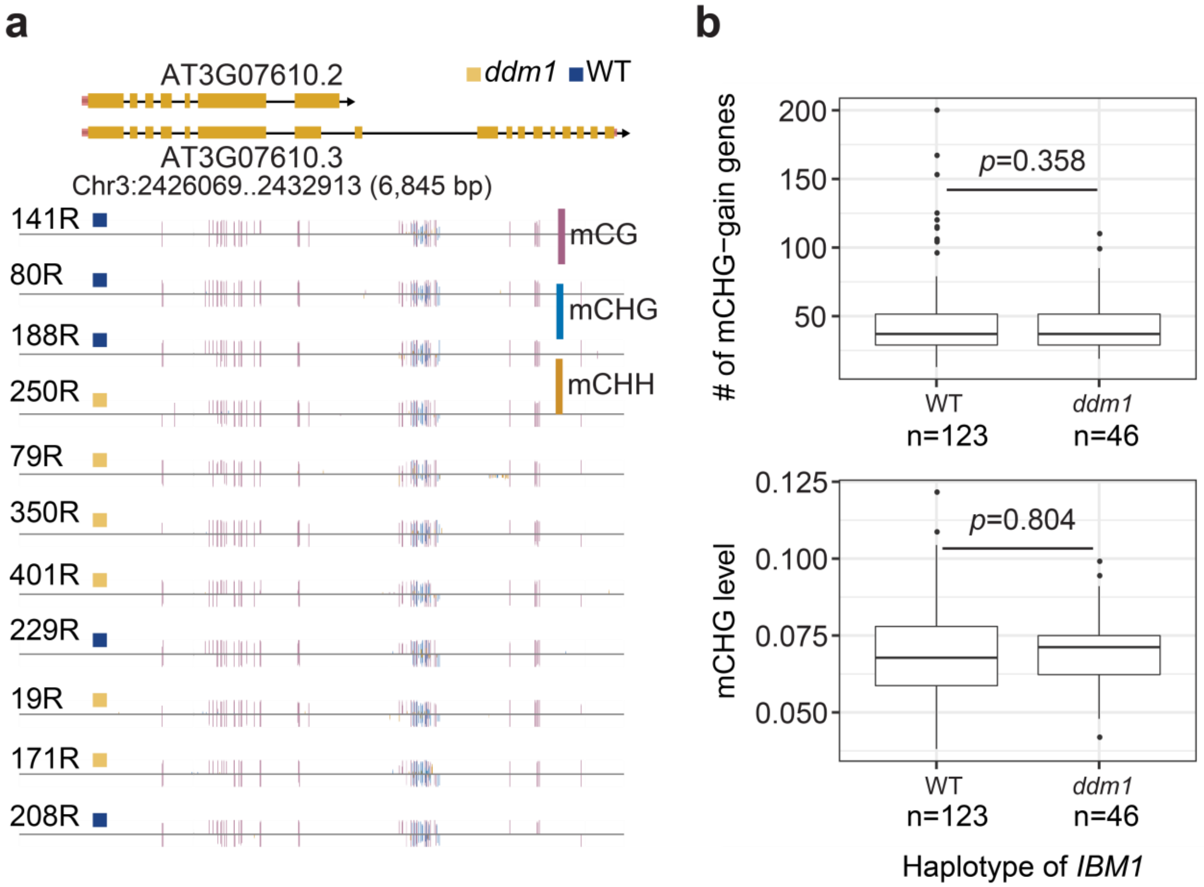

Supplementary Figure 4. Ectopic non-CG methylation was not influenced by intronic methylation of *IBM1* in *ddm1* epiRILs. a Mutations in the H3K9me2 demethylase, IBM1, results in ectopic non-CG methylation mainly on gbM genes. Functional expression of *IBM1* requires the presence of intronic methylation, the removal of which promotes expression of a truncated, non-functional transcript. However, no significant reduction of intronic methylation has been shown in *ddm1* epiRILs. **b** To further exploring the potential influence of *IBM1* on ectopic genic non-CG methylation, epiRILs were classified into two groups based on *IBM1* belonging to WT or *ddm1* haplotype, since *IBM1* inherited from a *ddm1* parent may suffer a reduction of intronic methylation. However, there are no significant difference between two groups in terms of the number of ectopic mCHG genes and genic mCHG level. P value is generated by two-sided student T-test. Box plots show a median center line, the lower and upper hinges are the first and third quartiles. Whiskers represent 1.5x the interquartile range. N=123 and 46 biologically independent samples are applied for two groups, respectively. Source data underlying Supplementary Figure 4b are provided as a Source Data file**.**

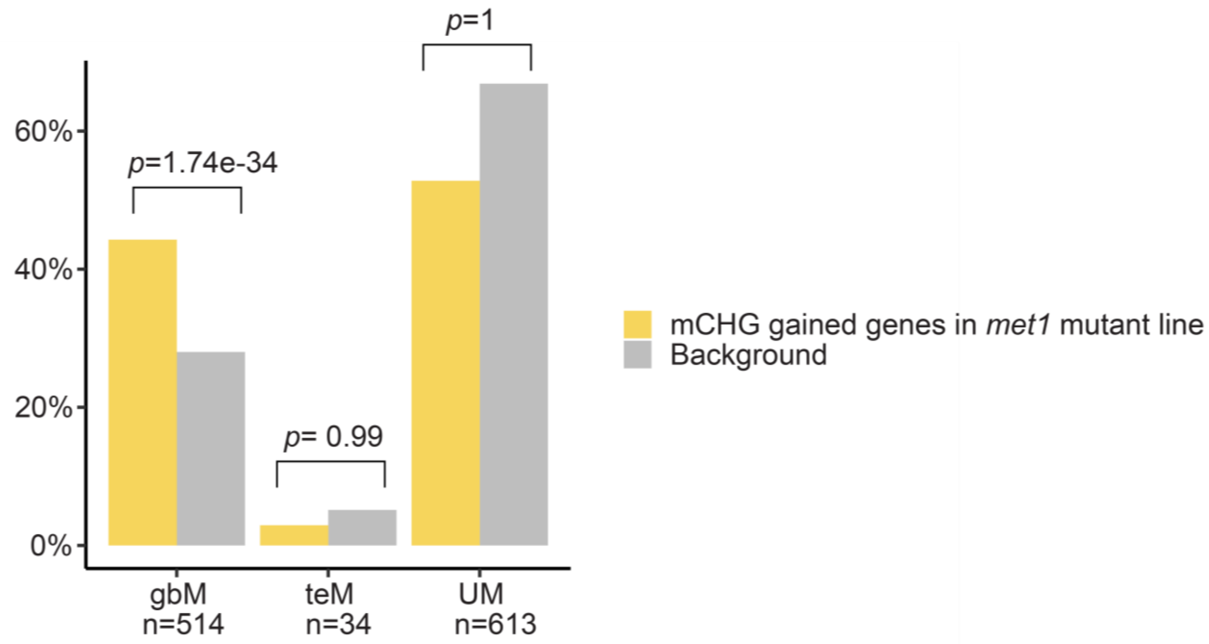

Supplementary Figure 5. Ectopic mCHG-gain genes in *met1* mutant are enriched in gbM genes. In total, there are 1161 mCHG-gain genes in *met1* mutant line. Based on an enrichment test, those genes are enriched in gbM genes. Yellow bars show the ratio of each type of genes with ectopic mCHG. Grey bars show the ratio of each type of genes in comparison with all coding genes. P value is based on one-sided Fisher's Exact enrichment test with alternative hypothesis that odds ratio is greater than 1. Source data are provided as a Source Data file**.**

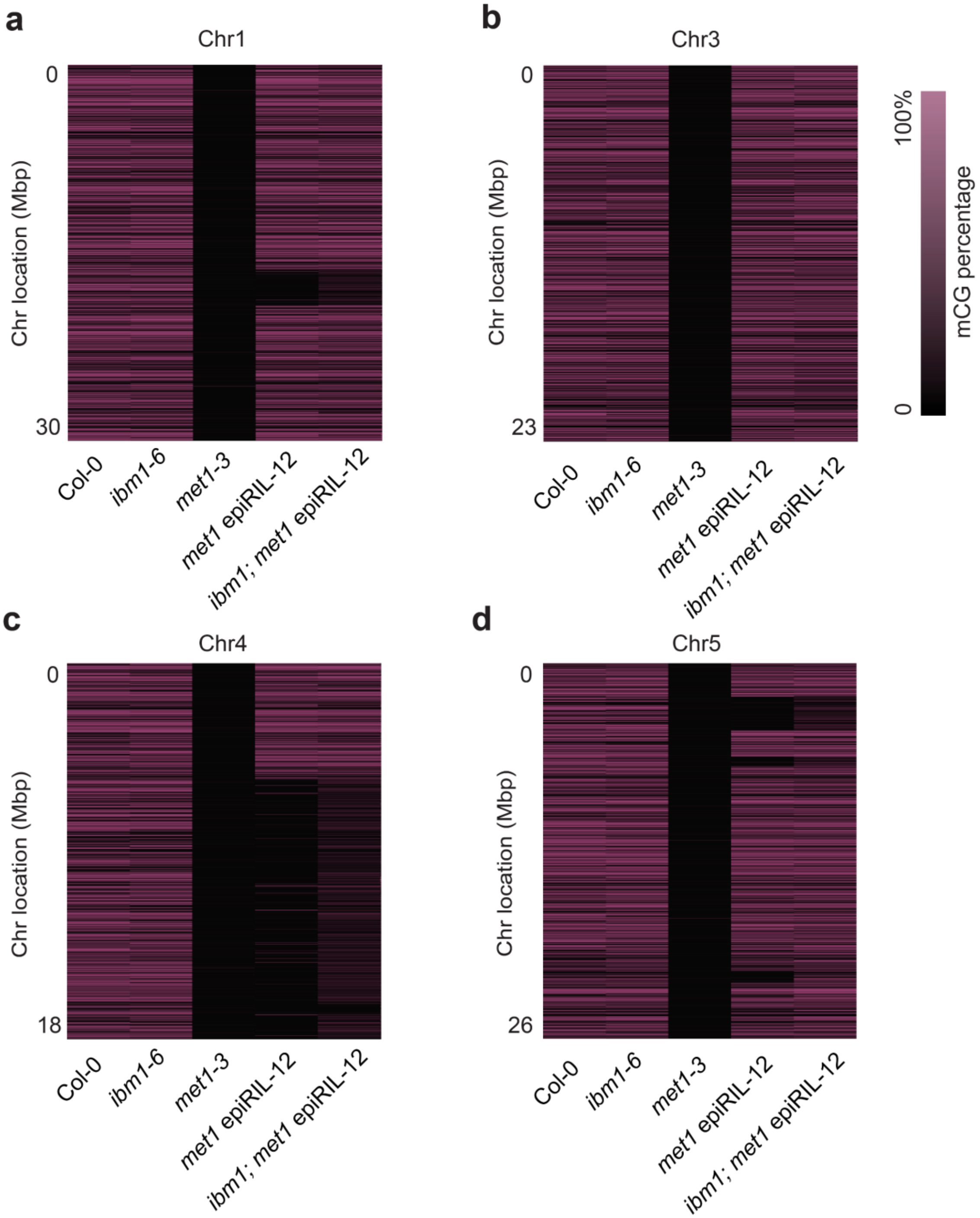

Supplementary Figure 6. Except chromosome 2, no obvious large region in *ibm1; met1 epiRIL-12 is* derived from the original *met1-3* parent. a Schematic plots show regions that inherited mCG from the *met1 epiRIL-12* parent or the *ibm1-6* parent on Chromosome 1 (*Col-0, ibm1-6* and *met1-3* are shown as controls).Purple to black colors show the mCG percentage of genes that possess gbM in Col-0 from 100% to 0%, respectively. **b** on Chromosome 3 **c** on Chromosome 4 **d** on Chromosome 5.

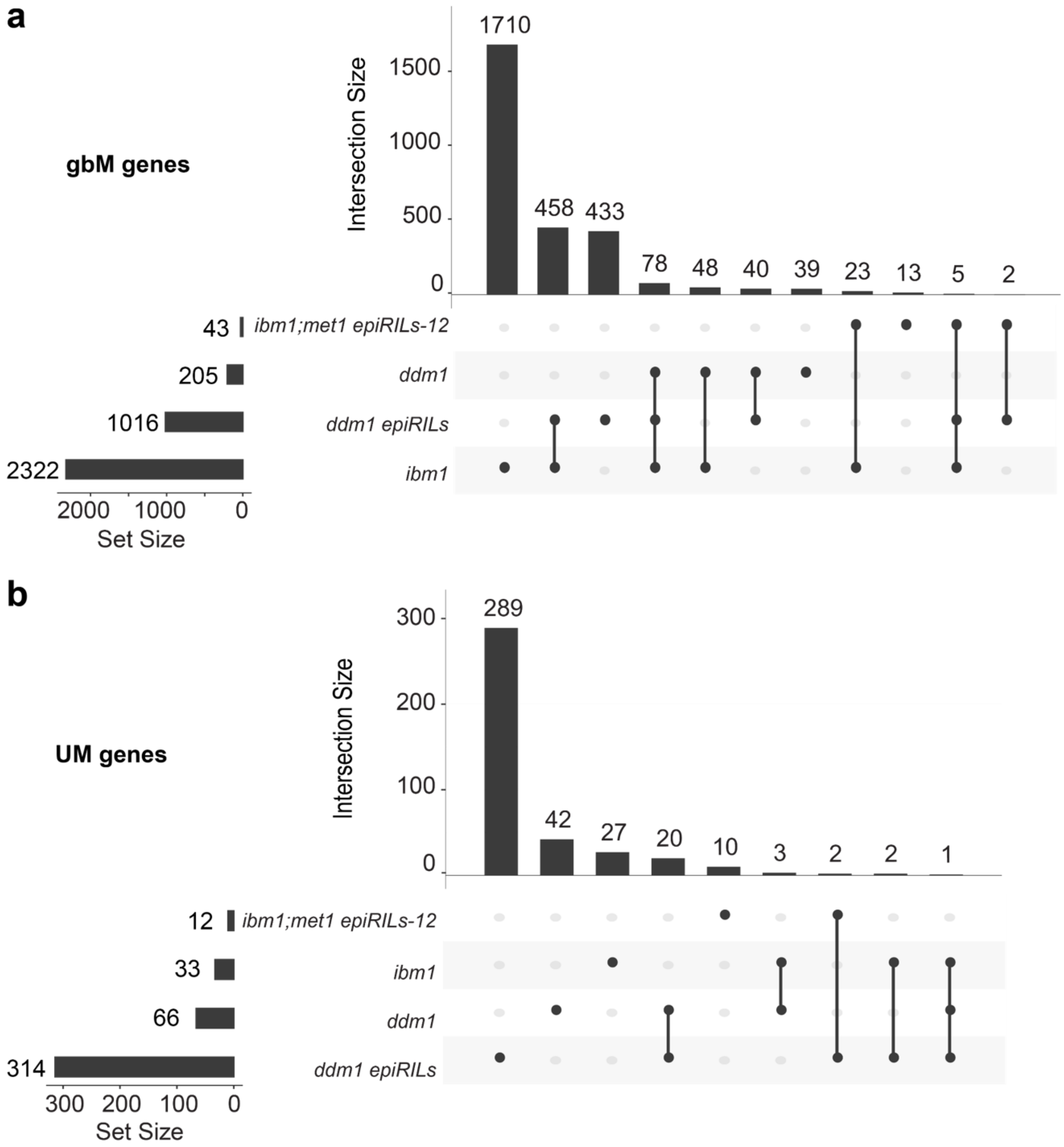

Supplementary Figure 7. Total size and intersections of ectopic mCHG-gain genes among four datasets in this study. a An Upset plot shows the total number of mCHG-gain gbM genes for each of four datasets (bar plot at the left side panel). Bars in the middle show the number of overlapped genes for each combination of datasets that indicated by linked black dots in the bottom panel. For bars with a single dot at bottom, it gives the number of genes that uniquely exist in a given dataset. **b** Same as above, but for ectopic mCHG happened on UM genes.

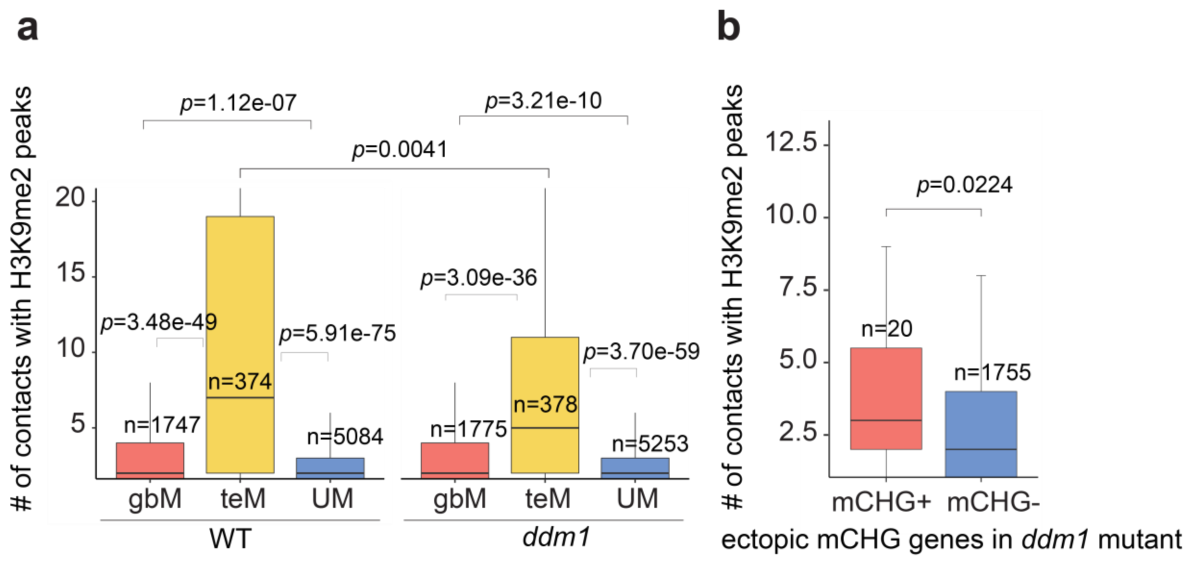

Supplementary Figure 8. Loci that are susceptible to spontaneous epiallele formation in *ddm1* mutant have a greater contact frequency with H3K9me2 region. a Distribution of the number of contacts between three types of genes (gbM, UM, teM) and H3K9me2 regions. Hi-C data for both wild-type (left panel) and *ddm1* mutant (right panel) were used for identifying significant interactions between genomic regions. **b** gbM genes were further classified into a group with ectopic mCHG (mCHG+) and a group without ectopic mCHG (mCHG-) based on *ddm1* 1G mutant line data. Distribution of the number of contacts between the two group of genes and H3K9me2 regions was generated based on Hi-C data of *ddm1* mutant. Two sided Wilcoxon tests was used for pairwise comparison of each two groups without adjustment for multiple comparisons. Box plots represented show a median center line, the lower and upper hinges are the first and third quartiles. Whiskers represent 1.5x the interquartile range. Source data are provided as a Source Data file.
