# Supplementary material for: Heterochromatin is a quantitative trait associated with spontaneous epiallele formation
Source: Nat Commun. 2021 Nov 29;12:6958. doi: 10.1038/s41467-021-27320-6 (PMC8630088; doi:10.1038/s41467-021-27320-6)
Supplement: Supplementary file 2 — Description of Additional Supplementary Files [file 41467_2021_27320_MOESM2_ESM.pdf]

Description of Additional Supplementary Files

File Name: Supplementary Data 1
Description: Summary of the MethylC-seq mapping results for 169 ddm1 epiRILs

File Name: Supplementary Data 2
Description: Summary of MethylC-seq mapping results and data source for *met1* and *ibm1* mutant line

File Name: Supplementary Data 3
Description: Methylation classes of coding genes in this research

File Name: Supplementary Data 4
Description: List of mCHG-gain genes in *ibm1* and *ddm1* mutant lines

File Name: Supplementary Data 5
Description: 144 stably inherited DMR regions in *ddm1* epiRILs.

File Name: Supplementary Data 6
Description: the number of genes with ectopic mCHG and the averaged mCHG level for each *ddm1* epiRIL line.

File Name: Supplementary Data 7
Description: List of mCHG-gain genes in *ddm1* epiRILs. mCHG-gain genes in epiRILs were determined based on the number of mCHG sites in a gene. The significance of mCHG-gain (*p*-value) was determined using a binomial distribution and then adjusted by BH FDR (*q*-value). A gene was treated as a mCHG-gain gene if it had a *q*-value <0.05, whereas it had a *q*-value >0.05 in wild type.

File Name: Supplementary Data 8
Description: Global genic mCHG related traits for methylation QTL analysis

File Name: Supplementary Data 9
Description: Identified QTL peaks for global traits

File Name: Supplementary Data 10
Description: Genic mCHG level on each mCHG gain gene which was used as a trait for methylation QTL analysis

File Name: Supplementary Data 11
Description: Identified QTL peaks for each mCHG-gain gene

File Name: Supplementary Data 12
Description: List of mCHG-gain genes in *met1-3* derived region. gbM and UM gene columns report total genes in the *met1-3* derived region on Chromosome 2.
