# Supplementary material for: Heterochromatin is a quantitative trait associated with spontaneous epiallele formation
Source: Nat Commun. 2021 Nov 29;12:6958. doi: 10.1038/s41467-021-27320-6 (PMC8630088; doi:10.1038/s41467-021-27320-6)
Supplement: Supplementary file 15 — Reporting Summary [file 41467_2021_27320_MOESM15_ESM.pdf]

nature portfolio

Corresponding author(s): Schmitz, Johannes

Last updated by author(s): Nov 7, 2021

Reporting Summary

Nature Portfolio wishes to improve the reproducibility of the work that we publish. This form provides structure for consistency and transparency in reporting. For further information on Nature Portfolio policies, see our Editorial Policies and the Editorial Policy Checklist.

Statistics

For all statistical analyses, confirm that the following items are present in the figure legend, table legend, main text, or Methods section.

n/a	Confirmed	
☐	☒	The exact sample size (*n*) for each experimental group/condition, given as a discrete number and unit of measurement
☐	☒	A statement on whether measurements were taken from distinct samples or whether the same sample was measured repeatedly
☐	☒	The statistical test(s) used AND whether they are one- or two-sided<br>*Only common tests should be described solely by name; describe more complex techniques in the Methods section.*
☒	☐	A description of all covariates tested
☒	☐	A description of any assumptions or corrections, such as tests of normality and adjustment for multiple comparisons
☐	☒	A full description of the statistical parameters including central tendency (e.g. means) or other basic estimates (e.g. regression coefficient) AND variation (e.g. standard deviation) or associated estimates of uncertainty (e.g. confidence intervals)
☐	☒	For null hypothesis testing, the test statistic (e.g. *F*, *t*, *r*) with confidence intervals, effect sizes, degrees of freedom and *P* value noted<br>*Give P values as exact values whenever suitable.*
☒	☐	For Bayesian analysis, information on the choice of priors and Markov chain Monte Carlo settings
☒	☐	For hierarchical and complex designs, identification of the appropriate level for tests and full reporting of outcomes
☐	☒	Estimates of effect sizes (e.g. Cohen's *d*, Pearson's *r*), indicating how they were calculated

Our web collection on statistics for biologists contains articles on many of the points above.

Software and code

Policy information about availability of computer code

Data collection	no software was used for data collection
Data analysis	Methylstar v1.4, Methimpute v1.16.0, Methylpy v1.3, cutadapt v1.9.devl, Rqtl v1.50, HiC-Pro v2.11.4, Fit-Hi-C v.2.0.7, Trim Galore v0.6.5, Sambamb v0.7.1, Bowtie v2.2.4, Bowtie2 v2.3.5.1, SAMtools v1. 10, MACS2. Customized scripts for methylome and mapping meQTL-epi loci analysis are available in (https://github.com/schmitzlab/Heterochromatin-quantitative-epiallele-formation)

For manuscripts utilizing custom algorithms or software that are central to the research but not yet described in published literature, software must be made available to editors and reviewers. We strongly encourage code deposition in a community repository (e.g. GitHub). See the Nature Portfolio guidelines for submitting code & software for further information.

Data

Policy information about availability of data

All manuscripts must include a data availability statement. This statement should provide the following information, where applicable:

- Accession codes, unique identifiers, or web links for publicly available datasets
- A description of any restrictions on data availability
- For clinical datasets or third party data, please ensure that the statement adheres to our policy

Previously published sequencing data used in this study are available as listed in Supplementary Data 2. WGBS data produced from this study have been deposited in the NCBI GEO database under GSE171157 [https://www.ncbi.nlm.nih.gov/geo/query/acc.cgi?acc=GSE171157] and GSE171414 [https://www.ncbi.nlm.nih.gov/geo/query/acc.cgi?acc=GSE171414].

Field-specific reporting

Please select the one below that is the best fit for your research. If you are not sure, read the appropriate sections before making your selection.

[x] Life sciences [ ] Behavioural & social sciences [ ] Ecological, evolutionary & environmental sciences

For a reference copy of the document with all sections, see nature.com/documents/nr-reporting-summary-flat.pdf

Life sciences study design

All studies must disclose on these points even when the disclosure is negative.

Sample size	Sample sizes were dependent on availability of ddm1 epiRILs as well as available funds for WGBS.
Data exclusions	No data was excluded from data analyses.
Replication	Replication was not performed for WGBS data. The propose of this research is not about finding the difference between control and treatment, but about exploring natural variation of DNA methylation in a group of biologically independent samples.
Randomization	No randomization of planting was performed. The purpose of this research is not about finding the difference between control and treatment, but about exploring natural variation of DNA methylation in a group of biologically independent samples.
Blinding	No blinding was performed.

Reporting for specific materials, systems and methods

We require information from authors about some types of materials, experimental systems and methods used in many studies. Here, indicate whether each material, system or method listed is relevant to your study. If you are not sure if a list item applies to your research, read the appropriate section before selecting a response.

Materials & experimental systems

n/a	Involved in the study
[x]	[ ] Antibodies
[x]	[ ] Eukaryotic cell lines
[x]	[ ] Palaeontology and archaeology
[x]	[ ] Animals and other organisms
[x]	[ ] Human research participants
[x]	[ ] Clinical data
[x]	[ ] Dual use research of concern

Methods

n/a	Involved in the study
[x]	[ ] ChIP-seq
[x]	[ ] Flow cytometry
[x]	[ ] MRI-based neuroimaging
